# Treatment of metastatic bladder paraganglioma with cadonilimab plus radiotherapy: a case report and literature review

**DOI:** 10.3389/fmed.2025.1718146

**Published:** 2026-01-22

**Authors:** RiHan Wu, ZiRui Wang, YuanRui Bai, Yihui Liu, Chunhui Dong, Ling Chen

**Affiliations:** 1 Department of Oncology, The First Affiliated Hospital of Xi'an Jiaotong University, Xi'an, China; 2 Department of Oncology, XD Group Hospital, Xi'an, China; 3 Health Science Center, Xi'an Jiaotong University, Xi'an, China; 4 Radiotherapy Oncology, People's Hospital of Ningxia Hui Autonomous Region, Yinchuan, China; 5 Cardiovascular Hospital, Ninth Hospital of Xi'an, Xi'an, China

**Keywords:** bladder paraganglioma, cadonilimab, case report, literature review, radiotherapy

## Abstract

**Background:**

Bladder paraganglioma (PPGL) is a rare neuroendocrine tumor associated with a lower survival rate compared to paragangliomas originating in other anatomical locations. Surgical resection is still the primary treatment modality for bladder paraganglioma; however, this approach carries a significant risk of malignant metastasis. Once metastasis occurs, therapeutic options become notably limited. The response rate and 5-year overall survival for bladder paraganglioma treated with cyclophosphamide, vincristine, and dacarbazine (CVD) chemotherapy are relatively low. Recent advancements in tumor immunotherapy, particularly antibodies targeting CTLA-4 and PD-1/PD-L1, have effectively treated various cancers, including neuroendocrine tumors. These immunotherapeutic approaches offer promising alternatives and potentially improve outcomes for patients with metastatic bladder paraganglioma.

**Case description:**

A 44-year-old male presented with intermittent hematuria, abdominal pain, and frequent urination. Pre-operative computed tomography (CT) revealed a neuroendocrine tumor and a laparoscopic partial cystectomy was subsequently performed. Post-operative pathology confirmed paraganglioma of the bladder, with immunohistochemical results showing SYN (+), P504S (+/–), and Ki-67 (+20%). The patient's post-operative symptoms resolved, returning to normal. However, recurrence occurred 33 months post-surgery. The patient later received 11 cycles of cadonilimab and local radiation therapy, with tumor progression evaluated via imaging every 2 months. During the follow-up period, the patient maintained stable disease for 12 months. After local progression, the patient received targeted therapy, chemotherapy, and palliative treatment. Survival time after recurrence is 25 months. Overall survival from diagnosis is 58 months.

**Conclusions:**

We report, for the first time, a case of metastatic bladder paraganglioma in which a patient treated with cadonilimab in combination with radiotherapy maintained stable disease for 12 months. These results demonstrate that this combination therapy may be a potential treatment option for bladder paraganglioma.

## Introduction

Pheochromocytomas and paragangliomas (PPGL) are rare endocrine tumors originating from neural crest-derived cells of the adrenal medulla or the sympathetic and parasympathetic nervous systems. Paragangliomas of the bladder, which arise from sympathetic nervous system chromaffin cells embedded in the bladder wall musculature, represent one of the rarest types of thoracoabdominal paragangliomas, accounting for approximately 0.06% of bladder tumors and 5% of paragangliomas (1, 2). Surgical resection is the primary treatment for bladder paraganglioma, but there is a significant risk of malignant metastasis. Metachronous metastasis occurs in 22% of patients with bladder paraganglioma, with a median onset of 4 years post-initial diagnosis, despite most patients undergoing surgical intervention (3). The optimal treatment options for metastatic bladder paraganglioma are currently limited. Guidelines recommend CVD regimens as the standard chemotherapy for metastatic PPGL, with 33% of patients responding to this treatment (4). However, this data pertains to PPGL in general, and the small sample size limits the prognostic data specific to bladder paraganglioma patients treated with the CVD regimen. Therefore, further research is required to identify effective treatment strategies for metastatic bladder paraganglioma patients who are not suitable for surgical treatment, to maintain disease stability and improve patient prognosis.

With the advancement of precision therapy, immunotherapy has emerged as a focal point in tumor treatment. Studies have demonstrated that some PPGLs can benefit from targeted PD-L1/2 therapy (5, 6). Compared to non-metastatic cases, metastatic PPGLs exhibit a higher proportion of PD-L1/2 immunopositivity (7). Cadonilimab (AK104) is a newly developed CTLA-4/PD-1 bispecific antibody. It has shown promise in several phase 1/2 clinical studies as a potential treatment for advanced solid tumors. Cadonilimab has been approved in China for the treatment of recurrent or metastatic cervical cancer that progresses during or after platinum-based chemotherapy and First-line treatment for locally advanced unresectable or metastatic gastric/gastroesophageal junction adenocarcinoma.

However, the efficacy of cadonilimab in treating bladder paraganglioma has not been documented. We present a case of metastatic paraganglioma of the bladder treated with a combination of cadonilimab and local radiotherapy.

## Case report

**Figure 1:**
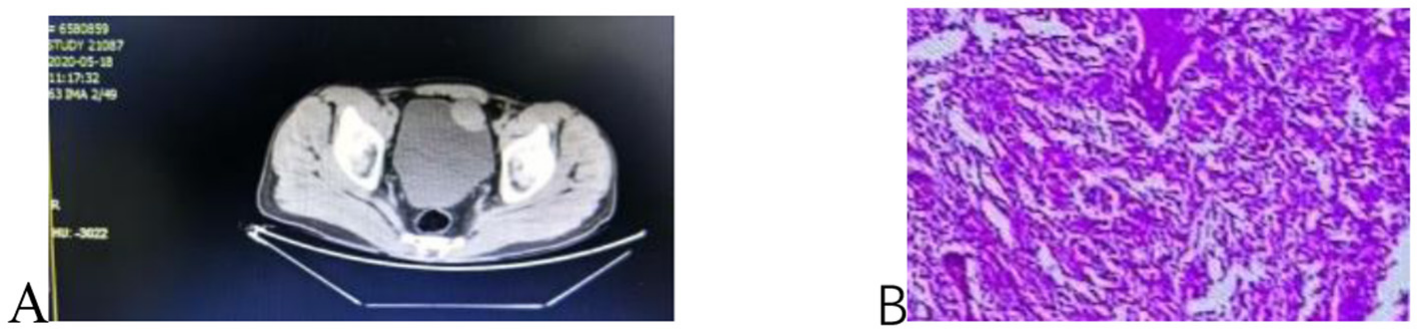
**(A)** Anterior bladder wall lesion **(B)** Immunohistochemical staining of bladder paraganglioma.

A 44-year-old male presented with intermittent hematuria, abdominal pain, and frequent urination, without accompanying symptoms of nausea, vomiting, hematemesis, melena, or fever, since January 2020. The patient had no chronic underlying diseases such as hypertension or diabetes, nor any history of psychological, genetic, or other significant illnesses. There was no family history of malignancy. He had a smoking history of over 20 years. He was seen at a local hospital on May 18, 2020. A pre-operative CT scan of the urinary system revealed a lump on the bladder's anterior wall, supplied by a branch of the left internal iliac artery, which was initially considered a neuroendocrine tumor (Figure 1A). The routine blood test, liver function test, and kidney function test results are normal. Urinalysis shows positive for occult blood. A laparoscopic partial cystectomy was performed at the local hospital. Immunohistochemistry (IHC) analysis showed positive staining for SYN, Ki-67 (20%), and P504S (+/–), while staining was negative for CKP, PSA, P63, EMA, P40, CD117, Dog-1, and CD34 (Figure 1B). Post-operatively, the patient's clinical symptoms resolved and returned to normal.

**Figure 2:**
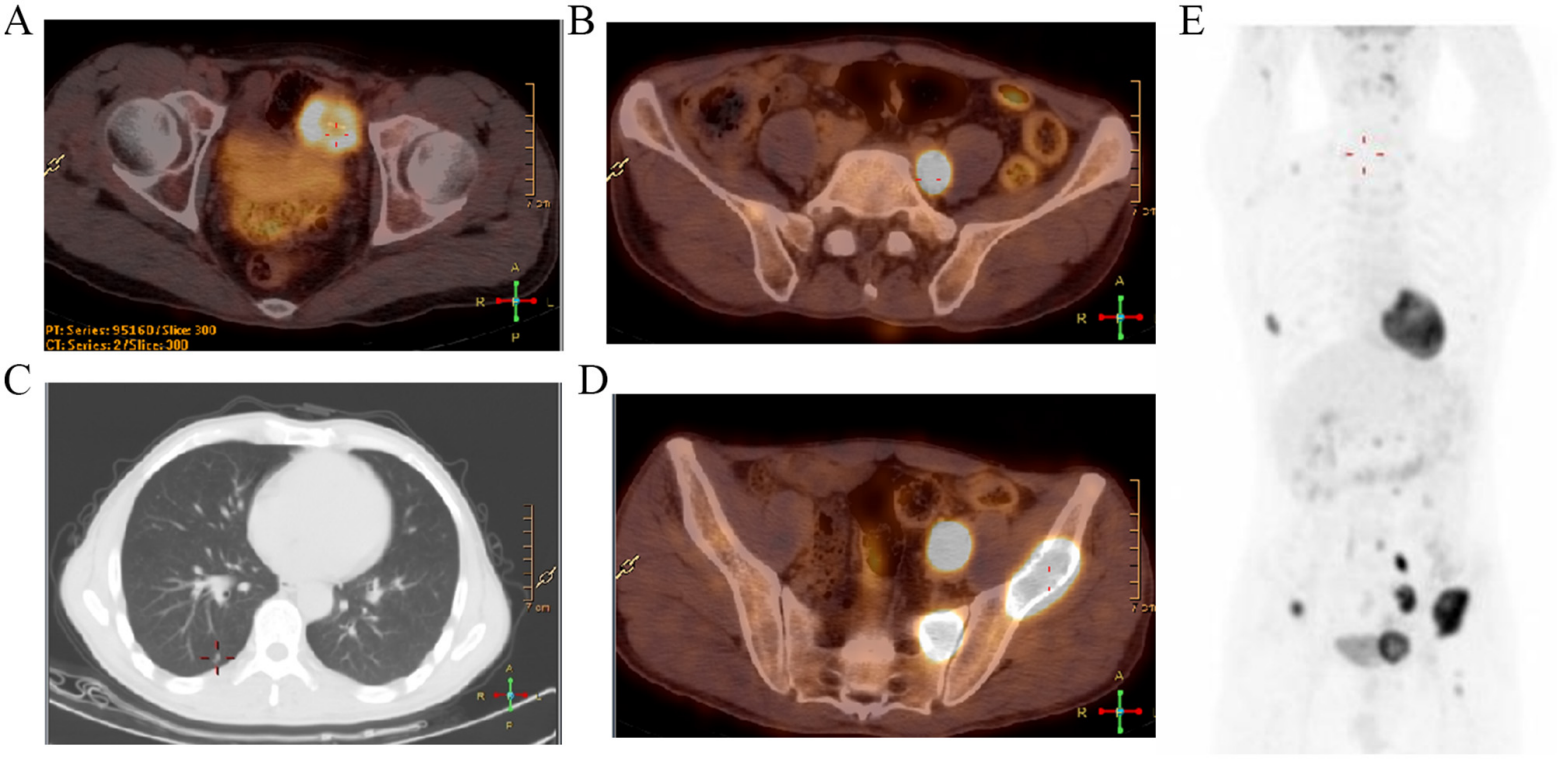
The results of CT/PET-CT. **(A–C)** High uptake localization of the left pelvis, retroperitoneal lymph nodes, and multiple micronodules in the lungs. **(D)** Osteolytic bone destruction and enhanced uptake in the pelvis and other sites. **(E)** Whole body PET-CT imaging.

**Figure 3:**
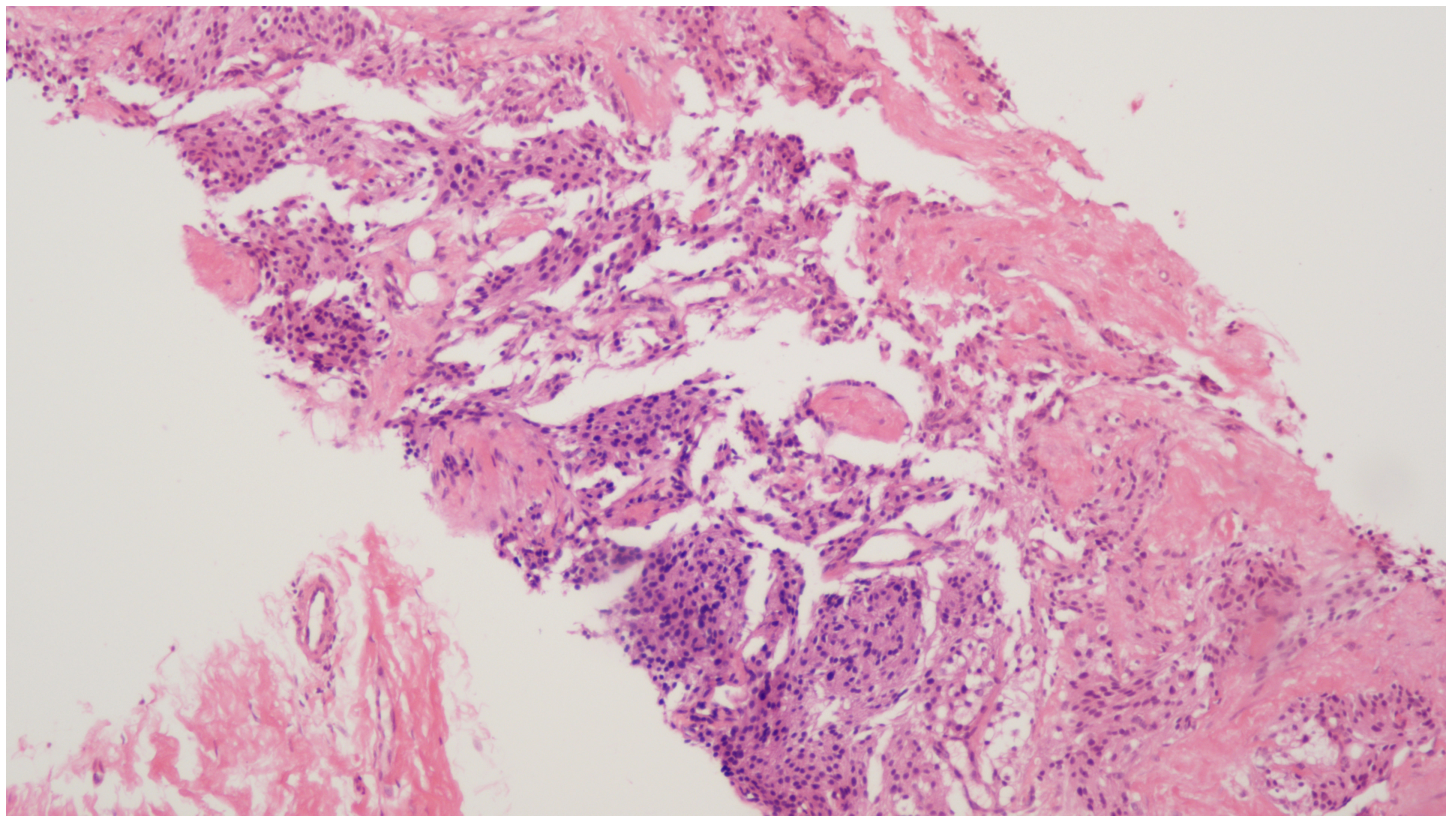
(HE × 10) Transperitoneal biopsy of anterior bladder mass: tumor tissue infiltration into fibrofatty tissue, and combined with immunohistochemical staining results suggests paraganglioma.

In September 2022, the patient again experienced intermittent hematuria, frequent urination, and urinary pain without obvious causes. He did not seek medical attention at that time. On February 16, 2023, due to recurrent symptoms, he was admitted to the Department of Urology at our hospital. Both the patient's clinical laboratory tests, including routine blood examination, serum electrolytes, and renal function, as well as his physical examination, returned normal results. The urinary endocrine biomarkers were as follows: urinary 24-h free epinephrine was 5.80 μg/24 h (normal range: 0–20 μg/24 h); urinary 24-h free norepinephrine was 1,175.65 μg/24 h (normal range: 0–90 μg/24 h); urinary 24-h free dopamine was 435.19 μg/24 h (normal range: 0–600 μg/24 h); urinary 24-h free methoxy adrenaline was 18.03 μg/24 h (normal range: 0–42.5 μg/24 h); urinary 24-h free methoxynorepinephrine was 2,208.81 μg/24 h (normal range: 0–57.1 μg/24 h); urinary 24-h free 3-methoxetamine was 293.33 μg/24 h (normal range: 0–63.8 μg/24 h); and urinary 24-h vanillylmandelic acid was 24.53 mg/24 h (normal range: 0–10.0 mg/24 h). Cystoscopy revealed a smooth mucosal bulge on the left side of the bladder following the resection of the bladder tumor. On February 24, 2023, PET-CT indicated a poorly demarcated soft tissue mass on the left side of the pelvis (41.3 mm × 40.7 mm × 36.4 mm) with a maximum standardized uptake value (SUVmax) of 18.2. The mass is poorly demarcated from the left wall of the bladder and adjacent intestinal loops. Multiple enlarged lymph nodes were observed around the left iliac vessels and in the retroperitoneum, with SUV max values of 21.5 and 5.5, respectively. Multiple nodules were observed in both lungs with an SUV max of 2.2, and multiple sites of bone destruction with increased radionuclide uptake were noted, showing an SUV max of 24.9 (Figure 2). A biopsy of the pelvic mass was performed on March 8, 2023, to confirm the diagnosis of the mass located in the bladder's left wall. Pathological examination confirmed the infiltration of tumor tissue into the fibrofatty tissue of the anterior space-occupying bladder. Immunohistochemical (IHC) staining suggested paraganglioma of the bladder, with positive results for Vim, CgA, Syn, CD56, GATA3 (weakly positive), negative results for CK, S100, PSA, P504S, Uroplakin, S100P, CK5/6, CK20, P63, NKX3.1, RCC, CAIX, and Ki67 (+40%; Figure 3). These findings confirmed that the patient had recurrent paraganglioma of the bladder with multiple systemic metastases.

**Figure 4:**
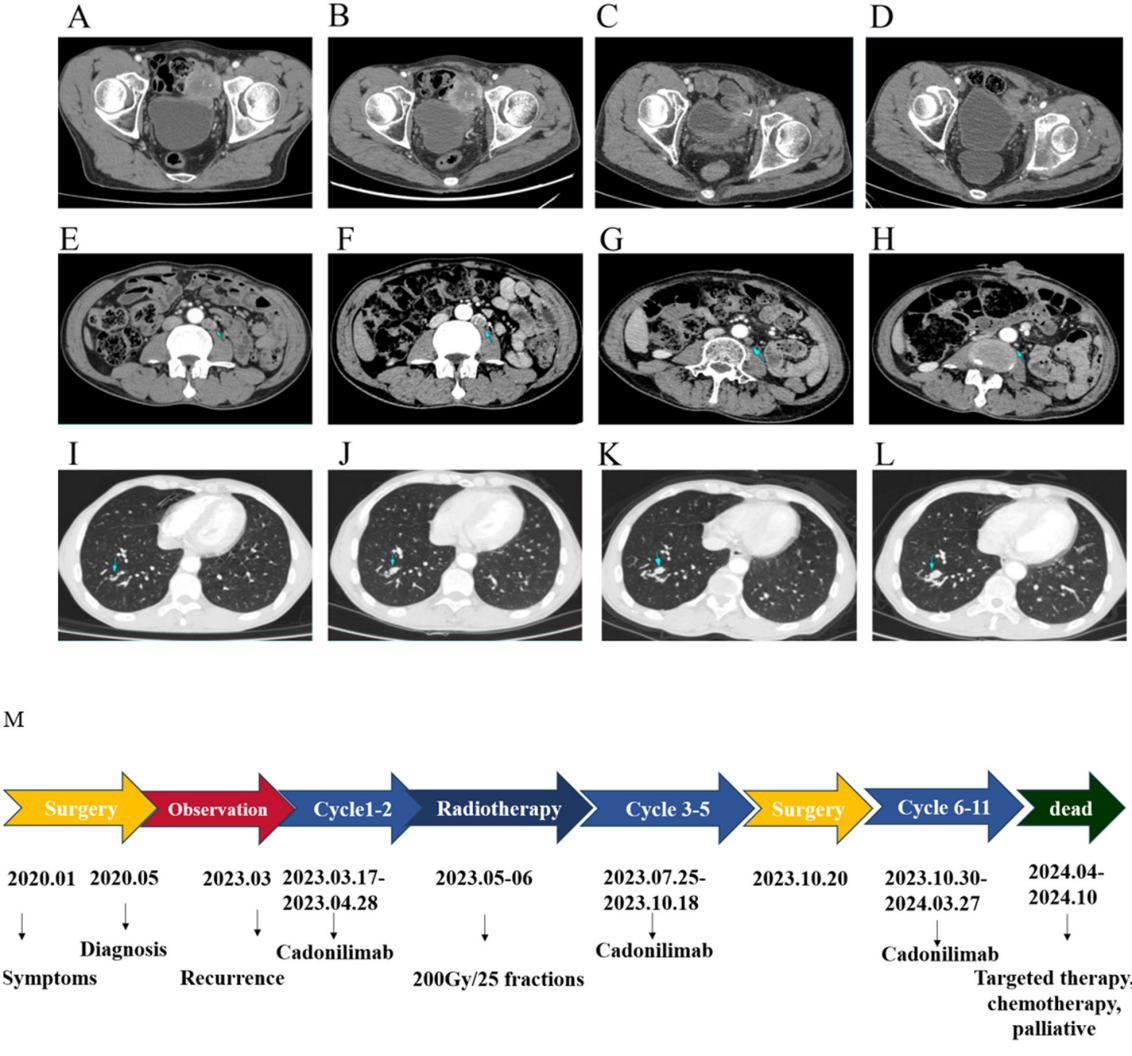
**(A–L)** The results of CT during radiotherapy plus 11 cycles of cadonilimab and the time flow diagram of the diagnostic and therapeutic process. The size of the pelvic mass and the metastatic lesion in the lymph node are gradually decreasing. The size of multiple micronodules had no significant progression. After two cycles of cadonilimab treatment, left pelvic mass 44 × 42 mm **(A, E, I)**, radiotherapy, left pelvic mass 44 × 47 mm **(B, F, J)**, eight cycles of cadonilimab treatment, left pelvic mass 43 × 46 mm **(C, G, K)**, and 10 cycles of cadonilimab treatment, left pelvic mass 36 × 34 mm **(D, H, L)**. **(M)** Patient treatment timeline.

The patient received cadonilimab treatment regularly once every 3 weeks from March 2023 to September 2023. The patient received local radiotherapy for bladder cancer in May 2023. He received a total of 5,000 cGy. The treatment was delivered in 25 fractions, 2Gy/fraction, over 5 weeks. Efficacy was evaluated according to the RECIST 1.1 criteria every two cycles, and the result was stable. On October 16, 2023, the patient was admitted to the hospital due to increased pain during urination and stool leakage. He was diagnosed with a colorectal fistula and underwent a double-tube transverse colostomy under general anesthesia on October 20, 2023. A CT scan on November 28, 2023, showed the left pelvic mass had decreased in size to 36 × 34 mm. The patient completed his 11th cycle of cadonilimab on March 6, 2024. The most recent CT scan on March 5, 2024, showed an irregular mass approximately 36 × 34 mm in size on the left side of the bladder (Figure 4). Mild skin itching was observed during follow-up. The patient achieved stable disease control for up to 1 year. The patient discontinued cadonilimab treatment due to personal reasons. After local progression, the patient received targeted therapy, chemotherapy, and palliative treatment at other hospitals. The patient died in October 2024. Survival time after recurrence is 25 months. Overall survival from diagnosis is 58 months.

## Discussion

The incidence of bladder paraganglioma is very low, accounting for 5% of paragangliomas and 0.06% of bladder tumors. The survival rate for bladder paraganglioma is lower compared to paragangliomas originating from other sites (8). Currently, the etiology of bladder paraganglioma is not clearly understood and may be related to genetic factors. Research over nearly 20 years has shown that approximately 40 percent of PPGL are genetically determined (9).

Surgical resection is the primary treatment for bladder paraganglioma. Common surgical procedures include partial cystectomy, radical cystectomy, and transurethral resection (TURBT) (10). Generally, the standard of care for muscularis-infiltrating tumors is partial cystectomy or radical cystectomy, while TURBT is an option for patients with small tumors confined to the submucosa. However, TURBT can cause an intraoperative hypertensive crisis due to sympathetic nervous system stimulation. Thus, adequate pre-operative preparation is essential to ensure patient safety. In this case, the patient underwent laparoscopic partial cystectomy, which offers the advantages of more complete tumor removal, reduced tumor stimulation, and bladder preservation. According to the most recent WHO categorization of neuroendocrine tumors, all paragangliomas (PGLs) have the capacity to metastasize, with 35%−40% of paragangliomas developing metastasis (11). Independent risk factors for PPGL metastasis include primary tumor size, a high Ki-67 index, an SDHB mutation, extra-adrenal location, and excess catecholamine production (3, 12, 13). Metachronous metastasis occurs in 22% of patients with bladder paraganglioma at a median of 4 years after the initial diagnosis, even when the majority of patients have been surgically treated. Once metastasis occurs, treatment options are limited. In this case, the patient was found to have recurrent bladder paraganglioma with multiple systemic metastases 33 months after surgery. Therefore, close post-operative follow-up is essential, and a strict monitoring plan should be established.

For patients with metastatic and inoperable bladder paraganglioma, systemic therapy can relieve clinical symptoms, control tumor growth, and prolong survival. Notably, there is currently no standard treatment for recurrent bladder paraganglioma. The latest guidelines from the Working Group on endocrine hypertension of the European Society of Hypertension recommend CVD regimen as first-line chemotherapy for rapidly progressive and radionuclide-treated slow- to moderate-growth PPGL (14). The National Institutes of Health followed 18 PPGL patients who received CVD chemotherapy over 22 years, including one patient with a primary tumor in the bladder. During follow-up, a partial response rate of 44% and a complete response rate of 11% were observed. Patients with tumors that responded to therapy had a median survival of 3.8 years, whereas those with non-reactive tumors had a median survival of 1.8 years (15). Other reports and meta-analyses indicate that patients with bladder paraganglioma can benefit from CVD regimens (16–18). Octreotide, a somatostatin analog (SSA), inhibits PPGL proliferation by binding to somatostatin receptor 2 (SSTR2). Wang et al. reported a case of metastatic bladder paraganglioma with post-operative recurrence, maintaining stable disease for 6 months after treatment with octreotide plus six cycles of CVD regimens (19). However, the tumor response rate and 5-year OS for bladder paraganglioma with CVD chemotherapy remain low. Moreover, CVD chemotherapy can cause severe side effects, such as myelosuppression. Radiotherapy, either alone or combined with other therapies, is another treatment option for metastatic bladder paragangliomas. A patient diagnosed with metastatic paraganglioma of the bladder underwent surgical resection, followed by a total of 55 Gy/25 fractions of radiation therapy to the entire pelvis over 4 weeks. Following up after 6 months, the patient exhibited no indications of recurrence (20). Additionally, advancements in nuclear medicine have shown that [131I]MIBG radiotherapy, based on SSTR, has a 63%−87% disease control rate. A Phase 2 trial involving 49 patients with metastatic pheochromocytoma and paraganglioma treated with high-dose [131I]MIBG radiation showed that 8% maintained disease stability over 12 months, while 35% experienced disease progression within 1 year of treatment. The estimated 5-year overall survival rate was 64% (21). Retrospective and Phase 2 studies of sunitinib showed disease control rates of 57 and 83%, respectively, with PFS of 4.1 and 13.4 months, respectively (4, 22). Furthermore, some PPGLs have shown benefit from targeted PD-L1/2 therapy (5, 6). Economides et al. (23) reported a case of metastatic paraganglioma treated with cabozantinib in combination with nivolumab. The proportion of PD-L1/2 immunopositivity in metastatic cases was higher than in non-metastatic cases, indicating that patients with metastatic PPGL are more likely to benefit from PD-1 inhibitors (7). In recent years, dual immunotherapy that targets PD-1 and CTLA-4 has gained attention. The efficacy of ipilimumab in combination with nivolumab for treating rare cancers was evaluated in a prospective Phase 2 study involving 32 patients with neuroendocrine tumors. The ORR was 44% in patients with high-grade neuroendocrine carcinoma (24). Additionally, Rodriguez et al. (25) reported a case of a patient with PPGL who was treated with nivolumab and ipilimumab. The patient's tumor demonstrated a continuous size reduction and remained stable for approximately 15 months (25). Therefore, immunotherapy presents a promising alternative to conventional chemotherapy for the treatment of bladder paraganglioma.

Bispecific antibody target two distinct antigens, thereby reducing the distance between tumor cells and immune cells, which enhances tumor cell killing. CTLA-4 inhibitors primarily act during the early stages of *T* cell development, whereas PD-1 inhibitors exert their effects at the effector stage following *T* cell maturation, often resulting in more sustained therapeutic effects. Cadonilimab is an Fc-null-designed, symmetric tetravalent antibody that is bispecific for CTLA-4 and PD-1. The toxicity of cadonilimab is significantly reduced by this design, which inhibits binding to Fc receptors and minimizes antibody-dependent cytotoxicity, cellular phagocytosis, and the production of interleukin-6 (IL-6) and IL-8 (26). Approved in China in June 2022 for the treatment of recurrent or metastatic cervical cancer after platinum-based chemotherapy, cadonilimab has demonstrated promise in treating advanced solid tumors (27). An objective response rate ranging from 16.7 to 32.3% and a manageable safety profile was reported in a multicenter phase 1b/2 study including 240 patients with advanced malignancies (28). Additionally, the efficacy of cadonilimab as a monotherapy or in conjunction with other therapies has been supported by several phase 1/2 studies (16, 29–33).

The treatment of this paraganglioma case is challenging, with the core difficulties lying in the high proliferative activity indicated by the elevated Ki-67 index, as well as the systemic tumor burden caused by multiple organ metastases. Both the patient and his family expressed a preference for utilizing the latest anti-tumor agents. After a thorough assessment of the patient's condition and discussions with the patient and family, we chose to administer cadonilimab which is known for its minimal adverse reactions. The patient received a total of 11 cycles of cadonilimab in conjunction with radiotherapy following recurrence. During the treatment process, the lesion regressed and then remained stable. The patient developed mild skin itching and urethrorectal fistula Itching is related to abnormal activation of *T* cells, release of inflammatory factors, impaired skin barrier function, and cross-reaction of immune cells with skin antigens. Due to mild itching, no special treatment is required. PET-CT examination before systemic treatment suggests that the tumor tissue was poorly demarcated from the left wall of the bladder and adjacent intestinal tract. A urethral rectal fistula appeared 7 months after treatment with cadonilimab and 5 months after radiotherapy. The first consideration for its formation is that the tumor itself invades to the urethra and the wall of the sigmoid colon. After treatment, rapid tumor regression may lead to local tissue defects. These defects disrupt the normal tissue barrier between the urethra and rectosigmoid, resulting in the formation of fistulas.

In addition, we also need to consider the correlation between the occurrence of urethral rectal fistula and direct tissue damage from local radiotherapy. During pelvic radiotherapy, even with precise targeting techniques, normal tissues in the pelvic floor area where the urethra and rectum are adjacent may be exposed to radiation. Radiation can damage the DNA of tissue cells and also injure local blood vessels, leading to impaired tissue blood supply, which in turn causes chronic inflammation and fibrosis. This damage does not appear immediately; it may gradually worsen months after radiotherapy, resulting in necrosis and thinning of the urethral and rectal wall tissues. When the damage to these two tissues breaches the mucosal layer and connects with each other, a fistula is formed. That is one of the more challenging long-term complications following pelvic radiotherapy.

Immune checkpoint inhibitors can activate the body's immune cells to attack tumors, they may also attack normal intestinal mucosa. This immune-related adverse event (irAE) can trigger inflammation, ulcers, and necrosis of the intestinal mucosa, disrupting the integrity of the intestinal wall. Safety and efficacy of nivolumab plus ipilimumab in patients with advanced non-clear cell renal cell carcinoma: results from the phase 3b/4 CheckMate 920 trial. Grade 3–4 immune-mediated AEs were diarrhea/colitis (7.7%) (34). Although the patient in this case used Cadonilimab, a PD-1/CTLA-4 bispecific antibody, diarrhea and enteritis were not observed, so it is not considered a major factor in the formation of urethrorectal fistulas.

It is worth noting that after treatment, patients need to be closely monitored for symptoms such as passage of feces during urination, frequent urination, urgency, and lower abdominal pain. Mild fistulas can be treated conservatively (fasting, gastrointestinal decompression, anti-infection therapy, and nutritional support), while severe fistulas usually require surgical intervention.

The patient achieved stable disease control for up to 1 year. To our knowledge, this represents the first case where bladder paraganglioma has been managed with a combination of cadonilimab and radiotherapy, offering valuable insights into potential treatment strategies for this rare disease. Especially for people with hypertension who cannot tolerate targeted therapy and chemotherapy. However, given that this study is based on a single case, further studies with longer durations and larger sample sizes are needed to verify the safety and efficacy of this treatment regimen as well as its synergistic effects with other therapies.

## Conclusion

In this report, we present the first documented case of a patient with metastatic bladder paraganglioma who achieved disease stability for up to 1 year following treatment with a combination of cadonilimab and radiotherapy. This case suggests that combining immunotherapy with radiotherapy may offer a promising alternative to conventional chemotherapy for managing bladder paraganglioma. It is also necessary to closely monitor the response to treatment in order to handle it promptly.

## Data Availability

The original contributions presented in the study are included in the article/supplementary material, further inquiries can be directed to the corresponding author.

## References

[1] Pastor-Guzmán JM López-García S Giménez-Bachs JS Ruíz-Mondejar R Cañamares-Pabolaza L Atiénzar-Tobarra M . Paraganglioma of the bladder: controversy regarding treatment. Urol Int. (2004) 73:270–5. doi: 10.1159/000080841 15539850

[2] Purnell S Sidana A Maruf M Grant C Agarwal PK. Genitourinary paraganglioma: demographic, pathologic, and clinical characteristics in the surveillance, epidemiology, and end results (SEER) database (2000–2012). Urol Oncol. (2017) 35:457. e9–457.e14. doi: 10.1016/j.urolonc.2017.02.006 PMC5476479 28325651

[3] Yu K Ebbehøj AL Obeid H Vaidya A Else T Wachtel H . Presentation, management, and outcomes of urinary bladder paraganglioma: results from a multicenter study. J Clin Endocrinol Metab. (2022) 107:2811–21. doi: 10.1210/clinem/dgac427 35882219 PMC9516048

[4] Ayala-Ramirez M Chougnet CN Habra MA Palmer JL Leboulleux S Cabanillas ME . Treatment with sunitinib for patients with progressive metastatic pheochromocytomas and sympathetic paragangliomas. J Clin Endocrinol Metab. (2012) 97:4040–50. doi: 10.1210/jc.2012-2356 22965939 PMC3683800

[5] Pinato DJ Black RJ Trousil S Dina RE Trivedi P Mauri F . Programmed cell death ligands expression in phaeochromocytomas and paragangliomas: relationship with the hypoxic response, immune evasion and malignant behavior. Oncoimmunology. (2017) 6:e1358332. doi: 10.1080/2162402X.2017.1358332 29147618 PMC5674959

[6] Vanova KH Uher O Meuter L Ghosal S Talvacchio S Patel M . PD-L1 expression and association with genetic background in pheochromocytoma and paraganglioma. Front Oncol. (2022) 12:1045517. doi: 10.3389/fonc.2022.1045517 36439433 PMC9691952

[7] Uher O Vanova KH Taïeb D Calsina B Robledo M Clifton-Bligh R . The immune landscape of pheochromocytoma and paraganglioma: current advances and perspectives. Endocr Rev. (2024) 45:521–52. doi: 10.1210/endrev/bnae005 38377172 PMC11244254

[8] Fischer T Gaitonde S Jones M Bandera B Goldfarb G. Anatomic location is the primary determinant of survival for paragangliomas. Am Surg. (2017) 83:1132–6. doi: 10.1177/000313481708301024 29391110

[9] Buffet A Burnichon N Favier J Gimenez-Roqueplo A. An overview of 20 years of genetic studies in pheochromocytoma and paraganglioma. Best Pract Res Clin Endocrinol Metab. (2020) 34:101416. doi: 10.1016/j.beem.2020.101416 32295730

[10] Li M Xu X Bechmann N Pamporaki C Jiang J Propping S . Differences in clinical presentation and management between pre- and postsurgical diagnoses of urinary bladder paraganglioma: is there clinical relevance? A systematic review. World J Urol. (2022) 40:385–90. doi: 10.1007/s00345-021-03851-x 34655306 PMC8921018

[11] Lam AK. Update on adrenal tumours in 2017 world health organization (WHO) of endocrine tumours. Endocr Pathol. (2017) 28:213–27. doi: 10.1007/s12022-017-9484-5 28477311

[12] Zhai H Ma X Nie W Li H Peng C Li X . Paraganglioma of the urinary bladder: a series of 22 cases in a single center. Clin Genitourin Cancer. (2017) 15:e765–71. doi: 10.1016/j.clgc.2017.03.010 28688872

[13] Nölting S Bechmann N Taieb D Beuschlein F Fassnacht M Kroiss M . Personalized management of pheochromocytoma and paraganglioma. Endocr Rev. (2022) 43:199–239. doi: 10.1210/endrev/bnab019 34147030 PMC8905338

[14] Lenders JWM Kerstens MN Amar L Prejbisz A Robledo M Taieb D . Genetics, diagnosis, management and future directions of research of phaeochromocytoma and paraganglioma: a position statement and consensus of the working group on endocrine hypertension of the European society of hypertension. J Hypertens. (2020) 38:1443–56. doi: 10.1097/HJH.0000000000002438 32412940 PMC7486815

[15] Huang H Abraham J Hung E Averbuch S Merino M Steinberg SM . Treatment of malignant pheochromocytoma/paraganglioma with cyclophosphamide, vincristine, and dacarbazine: recommendation from a 22-year follow-up of 18 patients. Cancer. (2008) 113:2020–8. doi: 10.1002/cncr.23812 18780317 PMC9094399

[16] Niemeijer ND Alblas G Hulsteijn LT Dekkers OM Corssmit EPM. Chemotherapy with cyclophosphamide, vincristine and dacarbazine for malignant paraganglioma and pheochromocytoma: systematic review and meta-analysis. Clin Endocrinol. (2014) 81:642–51. doi: 10.1111/cen.12542 25041164

[17] Stigliano A Lardo P Cerquetti L Aschelter AM Matarazzo I Capriotti G . Treatment responses to antiangiogenetic therapy and chemotherapy in nonsecreting paraganglioma (PGL4) of urinary bladder with SDHB mutation. Medicine. (2018) 97:e10904. doi: 10.1097/MD.0000000000010904 30045248 PMC6078645

[18] Yamauchi Y Matsukawa Y Kato M Yoshino Y YamamotoT Kobayashi H . A case of malignant paraganglioma of the urinary bladder treated with cyclophosphamide, vincristine, and dacarbazine chemotherapy and metaiodobenzylguanidine therapy. Nihon Hinyokika Gakkai Zasshi. (2018) 109:106–10. doi: 10.5980/jpnjurol.109.106 31006739

[19] Wang Z Liu F LiC Yuan H Xiang Y Wei C . Case report: octreotide plus CVD chemotherapy for the treatment of multiple metastatic paragangliomas after double resection for functional bladder paraganglioma and urothelial papilloma. Front Oncol. (2023) 12:1072361. doi: 10.3389/fonc.2022.1072361 36741690 PMC9895770

[20] Yoo KH Choi T Lee HL Song MJ Chung BI. Aggressive paraganglioma of the urinary bladder with local recurrence and pelvic metastasis. Pathol Oncol Res. (2020) 26:2827–9. doi: 10.1007/s12253-020-00841-z 32548698

[21] Gonias S Goldsby R Matthay KK Hawkins R Price D Huberty J . Phase II study of high-dose [131I]Metaiodobenzylguanidine therapy for patients with metastatic pheochromocytoma and paraganglioma. J Clin Oncol. (2009) 27:4162–8. doi: 10.1200/JCO.2008.21.3496 19636009 PMC2734428

[22] O'Kane GM Ezzat S Joshua AM Bourdeau I Leibowitz-Amit R Olney HJ . A phase 2 trial of sunitinib in patients with progressive paraganglioma or pheochromocytoma: the SNIPP trial. Br J Cancer. (2019) 120:1113–9. doi: 10.1038/s41416-019-0474-x 31105270 PMC6738062

[23] Economides MP Shah AY Jimenez C Habra MA Desai M Campbell MT. A durable response with the combination of nivolumab and cabozantinib in a patient with metastatic paraganglioma: a case report and review of the current literature. Front Endocrinol. (2020) 11:594264. doi: 10.3389/fendo.2020.594264 33329398 PMC7731902

[24] Patel SP Othus M Chae YK Giles FJ Hansel DE Singh PP . A phase II basket trial of dual anti-CTLA-4 and anti-PD-1 blockade in rare tumors (DART SWOG 1609) in patients with nonpancreatic neuroendocrine tumors. Clin Cancer Res. (2020) 26:2290–6. doi: 10.1158/1078-0432.CCR-19-3356 31969335 PMC7231627

[25] Rodriguez RR Rizwan S Alhamad K Finley GG. The use of immunotherapy treatment in malignant pheochromocytomas/paragangliomas: a case report. J Med Case Rep. (2021) 15:172. doi: 10.1186/s13256-021-02733-5 33775249 PMC8006354

[26] Pang X Huang Z Zhong T Zhang P Wang ZM Xia M . Cadonilimab, a tetravalent PD-1/CTLA-4 bispecific antibody with trans-binding and enhanced target binding avidity. MAbs. (2023) 15:2180794. doi: 10.1080/19420862.2023.2180794 36872527 PMC10012886

[27] Keam SJ. Cadonilimab: first approval. Drugs. (2022) 82:1333–9. doi: 10.1007/s40265-022-01761-9 35986837

[28] Gao X Xu N Li Z Shen L Ji K Zheng Z . Safety and antitumour activity of cadonilimab, an anti-PD-1/CTLA-4 bispecific antibody, for patients with advanced solid tumours (compassion-03): a multicentre, open-label, phase 1b/2 trial. Lancet Oncol. (2023) 24:1134–46. doi: 10.1016/S1470-2045(23)00411-4 37797632

[29] Frentzas S Gan HK Cosman R Coward J Tran B Millward M . A phase 1a/1b first-in-human study (compassion-01) evaluating cadonilimab in patients with advanced solid tumors. Cell Rep Med. (2023) 4:101242. doi: 10.1016/j.xcrm.2023.101242 37852261 PMC10694581

[30] Qiao Q Han C Ye S Li J Shao G Bai Y . The efficacy and safety of cadonilimab combined with lenvatinib for first-line treatment of advanced hepatocellular carcinoma (compassion-08): a phase Ib/II single-arm clinical trial. Front Immunol. (2023) 14:1238667. doi: 10.3389/fimmu.2023.1238667 37942328 PMC10627805

[31] Chen B Yao W Li X Lin G Chu Q Liu H . A phase Ib/II study of cadonilimab (PD-1/CTLA-4 bispecific antibody) plus anlotinib as first-line treatment in patients with advanced non-small cell lung cancer. Br J Cancer. (2024) 130:450–6. doi: 10.1038/s41416-023-02519-0 38110665 PMC10844309

[32] Lou H Cai H Huang X Li G Wang L Liu F . Cadonilimab combined with chemotherapy with or without bevacizumab as first-line treatment in recurrent or metastatic cervical cancer (compassion-13): a phase 2 study. Clin Cancer Res. (2024) 30:1501–8. doi: 10.1158/1078-0432.CCR-23-3162 38372727 PMC11016896

[33] Chen QY Guo SS Luo Y Qu S Wu DH Chen XZ . Efficacy and safety of cadonilimab in previously treated recurrent or metastatic nasopharyngeal carcinoma (compassion-06): a phase II multicenter study. Oral Oncol. (2024) 151:106723. doi: 10.1016/j.oraloncology.2024.106723 38387261

[34] Tykodi SS Gordan LN Alter RS Arrowsmith E Harrison MR Percent I . Safety and efficacy of nivolumab plus ipilimumab in patients with advanced non-clear cell renal cell carcinoma: results from the phase 3b/4 CheckMate 920 trial. J Immunother Cancer. (2022) 10:e003844. doi: 10.1136/jitc-2021-003844 35210307 PMC8883262

